# Do epinephrine auto-injectors have an unsuitable needle length for young children?

**DOI:** 10.1186/1710-1492-10-S1-A19

**Published:** 2014-03-03

**Authors:** Laura Kim, Immaculate FP Nevis, Gina Tsai, Arunmozhi Dominic, Ryan Potts, Jack Chiu, Harold Kim

**Affiliations:** 1 Department of Anatomy and Cell Biology, McGill University, Montreal, Quebec, Canada, H3A 0G4; 2 Michael DeGroote School of Medicine, McMaster University, Hamilton, Ontario, Canada, L8S 4L8; 3 Schulich School of Dentistry and Medicine, Western University, London, Ontario, Canada, N6A 3K7; 4 Department of Biology, University of Waterloo, Waterloo, Ontario, Canada, N2L 3G1

## Background

Epinephrine delivered by an auto-injector to the anterolateral aspect of the thigh is the standard of care for the emergency treatment of anaphylaxis. For most pediatric patients in Canada, the EpipenJr® is prescribed, which has a needle length of 12.7mm. The route of epinephrine administration affects its onset of action, and intramuscular delivery is recommended for rapid absorption. If epinephrine is injected subcutaneously, the absorption will be slower. Conversely, if it is injected into the bone, the absorption will be unpredictable. There are no published clinical studies assessing whether the needle length of the EpipenJr® is adequate to deliver epinephrine intramuscularly in pediatric patients at risk of anaphylaxis.

## Methods

Consecutive pediatric patients under 15kg with confirmed food allergy who required prescriptions or refills of EpipenJr® at an allergist’s office were included in this study. An ultrasound of the anterolateral aspect of the mid thigh was performed under minimal (min) and maximal (max) pressure. Measurements of skin-to-muscle depth (STMD) and skin-to-bone depth (STBD) were completed. Baseline characteristics between two patient groups were compared: patients with STBD_max_ less than 12.7mm and patients with STBD_max_ greater than or equal to 12.7mm. Multivariable linear regression was performed including variables such as age, sex, BMI and race. The likelihood of the STBD_max_ of less than 12.7 mm was calculated for the weight groupings of <9kg,<11kg and <15kg.

## Results

A total of 75 participants were included in this study. There were 21 patients (28%) that had STBD_max_ less than 12.7mm. Baseline characteristics differed significantly for height and weight of the participants between the two groups (p<0.05). Multivariable linear regression showed that age (p=0.0002) and BMI (p=0.00008) were significantly associated with STBD_max_, following adjustment for sex and race. For patients under 9kg, 90% had STBD_max_ less than 12.7mm. For patients under 11kg, 53% had STBD_max_ less than 12.7mm.

## Conclusions

Based on this study, there are a significant number of children under 15kg at risk of receiving an epinephrine auto-injector into the bone. Because of this risk, epinephrine auto-injectors should be prescribed with caution in this population.

